# Uniaxial and Coaxial Nanofibers PCL/Alginate or PCL/Gelatine Transport and Release Tamoxifen and Curcumin Affecting the Viability of MCF7 Cell Line

**DOI:** 10.3390/nano12193348

**Published:** 2022-09-26

**Authors:** Diego Fernando Suárez, Ana Delia Pinzón-García, Rubén Darío Sinisterra, Anderson Dussan, Fredy Mesa, Sandra Ramírez-Clavijo

**Affiliations:** 1 Chemistry Department, Instituto de Ciências Exatas, Universidade Federal de Minas Gerais, Av. Presidente Antônio Carlos 6627, Belo Horizonte 31270-901, MG, Brazil; 2 Departamento de Física, Grupo de Materiales Nanoestructurados y sus Aplicaciones, Universidad Nacional de Colombia, Bogotá 110011, Colombia; 3 Department of Biology, Grupo Ciencias Básicas Médicas, Faculty of Natural Science, Universidad del Rosario, Bogotá 110311, Colombia

**Keywords:** nanofiber, curcumin, tamoxifen, breast cancer, electrospinning, MCF-7

## Abstract

Breast cancer is the second cause of cancer death in women worldwide. The search for therapeutic and preventive alternatives has increased in recent years. One synthetic drug for patients with hormone receptor-positive tumours is tamoxifen citrate (TMX). Curcumin (Cur) is a natural compound that is being tested. Both were coupled with nanoscale-controlled and sustained release systems to increase the effectiveness of the treatment and reduce adverse effects. We produced a controlled release system based on uniaxial and coaxial polymeric nanofibers of polycaprolactone (PCL), alginate (Alg) and gelatine (Gel) for the transport and release of TMX and Cur, as a new alternative to breast cancer treatment. Nanofibers combining PCL–Alg and PCL–Gel were fabricated by the electrospinning technique and physicochemically characterised by thermal analysis, absorption spectroscopy in the infrared region and X-ray diffraction. Morphology and size were studied by scanning electron microscopy. Additionally, the release profile of TMX and Cur was obtained by UV-Vis spectroscopy. Additionally, the cytotoxic effect on breast cancer cell line MCF7 and peripheral-blood mononuclear cells (PBMCs) from a healthy donor were evaluated by a Resazurin reduction assay. These assays showed that PCL–TMX nanofiber was highly toxic to both cell types, while PCL–Cur was less toxic.

## 1. Introduction

Cancer is a group of multifactorial diseases, caused by the accumulation of mutations in DNA, which leads cells to express a genetic program that establishes internal and external communication networks promoting proliferation, invasion of other tissues and establishment of new growth foci [1]. The sustained increase in the prevalence of cancer in the world population is associated with aging and the presence of socioeconomic risk factors, which is why it is of concern to the World Health Organization (WHO). The International Agency for Research on Cancer (IARC) publishes data collected from cancer patient registries in various countries on the web and in the GLOBOCAN bulletin. According to this report, one in five people develop some type of cancer during their lifetime, and the global burden of cancer was estimated at 9.9 million deaths and 19.3 million new cases in the world in 2020 [2], while it is predicted that by 2030, there will be 13.2 million deaths and 21.4 million new cases [3]. In women, the most frequent type of cancer is breast cancer, which represents 11.7% of total cancer incidences and 6.9% of its mortality [2]. This disease is highly morbid and associated with multiple risk factors, such as genetic predisposition [4], oestrogen exposure [5], unhealthy diet [6], reproductive characteristics [7] and the presence of obesity [8]. The therapy applied to the patient depends on the subtype of cancer and the stage of the disease at the time of diagnosis, either pre-operative (neoadjuvant) or post-operative (adjuvant) or both. Male or female patients with oestrogen receptor (ER)-positive and ERBB2-negative breast tumours [9], after surgery to remove the tumour, are treated with adjuvant therapy with the anti-estrogenic drug tamoxifen citrate (TMX), intended to selectively block the ER. TMX is recommended as an adjuvant in women who have not reached menopause, for a period of 5 to 10 years [4,10], while other drugs with a similar effect include letrozole, anastrozole or exemestane [11,12,13]. TMX inhibits the proliferation of malignant cells and induces their death [14,15]. Thus, in women who have had ductal carcinoma, it reduces the risk of presenting a bilateral lesion by 50% and of presenting metastases by 40–50%, and it also reduces recurrence by 30–50% in pre-menopausal women. In healthy women at a high risk of acquiring the disease, the use of TMX as prophylaxis reduces the occurrence of breast cancer by 38% [16]. TMX has an antagonistic or agonistic effect on ER, depending on the organ [2]. It is agonistic in the liver, uterus and bones, and antagonistic in the brain and mammary glands [14,15]. In the search to reduce the adverse or collateral effects of systemic therapies, clinical trials with natural and/or phytotherapeutic compounds are being evaluated for the treatment of breast cancer because they present promising mechanisms of action (chemopreventive and therapeutic), since they act as regulators of the expression of genes involved in cell growth, tumour progression and metastasis. Among the most promising molecules are green tea polyphenols, genistein, resveratrol, sulforaphane and curcumin [17], which act by modulating epigenetic mechanisms via inhibitors of histone deacetylases (HDAC) and DNA methyltransferases (DNMT), and are, therefore, called epi-drugs [18]. Curcumin (Cur) is the common name of the active principle of the roots of the turmeric plant (Curcuma longa), which, in India, is used as a condiment [19,20] and is in traditional medicine [21].

It has a protective effect against cancers of the skin [22], oral cavity, pancreas and intestine, as well as acting as an antioxidant, bactericidal, anti-arthritic, anti-amyloid, anti-ischemic and anti-inflammatory agent [19,23]. Studies with breast cancer cell lines, such as MDA-MB-231, BT-483 and MCF7, among others, have revealed that its anticancer effect targets components of signalling pathways, such as the Wnt/catenin pathway [24]. It also inhibits cell proliferation by deregulating NF-kappa beta, cyclin D and metalloprotease-1 factors, and induces tumour cell death by activating intrinsic and extrinsic apoptosis pathways [25]. It promotes the increase in reactive oxygen species (ROS) that modify the permeability of the mitochondrial membrane in order to activate apoptosis, activates caspase 3 and promotes the expression of p21 protein, an important inhibitor of proliferation and a promoter of apoptosis [24]. Curcumin induces senescence and inhibits not only metastasis, but also inflammation and angiogenesis [23,26]. Other studies in MCF-7, MDA-MB-435 and MDA-MB-231 found that it induces upregulated or downregulated miRs and lcnRNAs in lung, colorectal, prostate, nasopharyngeal, pancreatic, leukaemia, ovarian and breast cancer [27,28]. Curcumin is highly hydrophobic and has phenyl groups that hinder the processes of cellular absorption and transport, giving the molecule low bioavailability. To treat breast cancer, it dissolves in polar solvents such as ethanol, methanol, chloroform and acetonitrile [29] and binds to nanocarrier devices with formulations based on curcumin-cyclodextrin inclusion compounds with alginate and polyethylene-glycol [20,30,31], which are used in photodynamic therapy. They are also used in dressings for the treatment of skin wounds [32]. Other types of carriers include exosomes [28,33] and liposomes [34]. Many chemotherapeutic agents approved by the FDA [34] possess little or no selectivity for tumour cells, resulting in severe side effects. Moreover, some of them are inactivated by resistance mechanisms that expel them out of the cell before they can reach their target [35,36]. For these reasons, the development of more effective treatment systems, with greater selectivity and a lower likelihood of generating resistance, is crucial. Controlled release systems based on nanofibers are of great interest because at a specified time, adequate drug concentrations can be reached in a specific site within the organism, causing a desired therapeutic effect and reducing side effects.

Nanofibers are a versatile tool because their composition, size and shape can be controlled, as well as making surface modifications, in order to minimise toxic potential and improve the distribution in the body, ensuring a safe and effective delivery vehicle. They are biocompatible, biodegradable and excellent support matrices. Prior to clinical use, the release and degradation time, as well as their biological activity, can be known. The drug is encapsulated and isolated from the external environment by a physical barrier that, by enzymatic or non-enzymatic mechanisms, dissolves and disintegrates when in contact with biological fluids, favouring the release of the active agent that it transports [37,38]. Furthermore, the drug is transported to the tissue or to the target within the organism. This system protects the drug against degradation, increases its circulation time, reduces immunogenicity and toxicity, and increases specificity and selectivity, among other benefits [31,39]. Nanofibers are manufactured based on polymer solutions, both synthetic and natural, alone or combined, using different procedures, among which the electrospinning technique is the most widely used for applications in medicine [40]. With this technique, continuous ultrafine fibres with diameters in the micro-nanometre range are obtained from polymeric solutions or melted polymers by applying an electric field [31,39]. They are made from synthetic polymers, for example, polycaprolactone (PCL), poly-lactic/poly-glycolic acid (PLGA), poly-vinyl alcohol (PVA), poly-lactic acid (PLA), polyvinylpyrrolidone (PVP), and natural polymers such as alginate, gelatine, collagen, hyaluronic acid, elastin, etc. [41,42,43,44]. Poly-ε-caprolactone (PCL) is most frequently used in the manufacture of controlled release systems [30]. It is a semi-crystalline, biodegradable and biocompatible aliphatic polyester, which is obtained by the ring-opening reaction of ε-caprolactone, using methods such as phase separation, self-organization, polymerization, electrospinning, among others [40]. It has been used as a release vehicle containing curcumin alone or embedded in gelatine [45]. However, there is no report of uniaxial and coaxial nanofibers with the PCL–Alg or PCL–gelatine combination with cytotoxicity tests on PBMCs and the MCF7 breast cancer cell line.

In this study, nanofibers were manufactured by electrospinning uniaxial and coaxial PCL with two hydrosoluble polymers in order to evaluate the effect of the coating on the release of curcumin, as well as evaluating nanofibers loaded with tamoxifen as a control compound for assessing substance release systems and an anticancer effect. They were characterized by SEM, FTIR, XRD and the thermogravimetric method; on the other hand, the cytotoxicity was evaluated in a MCF7 breast cancer cell line and PBMCs by a Resazurin reduction assay.

## 2. Materials and Methods

### 2.1. Materials

PCL (*M_w_* = 43,000–50,000 g/mol) and tamoxifen citrate were purchased from Polysciences, Inc. (Warrington, PA, USA) and Drogaria Araujo S.A. (Belo Horizonte, Brazil), respectively. Gelatine (bovine skin type B), sodium alginate, curcumin, glacial acetic acid (99.9%) and ethyl acetate (anhydrous, 99.8%) were obtained from Sigma-Aldrich (São Paulo-SP, Brazil). Dichloromethane (DCM) and methanol (MET) were purchased from Vetec (São Paulo-SP, Brazil). All other materials used were analytical grade.

### 2.2. Preparation of PCL Nanofiber Solutions and Electrospinning

Uniaxial and coaxial polymeric fibres of polycaprolactone (PCL), alginate (Alg) and gelatine (Gel) were prepared at the following concentrations: PCL at 10% *w*/*v*, Alg at 0.1% *w*/*v* and Gel solution at 8% *w*/*v*. The uniaxial nanofibers were loaded with curcumin and tamoxifen as active substances, and the coaxial nanofibers were loaded with curcumin. Table 1 describes the preparation conditions of each of the fibres used in this study, where uniaxial PCL fibres loaded separately with Cur and TMX were prepared using 10 mL of 10% *w*/*v* PCL solution in a 1:1 methanol–dichloromethane mixture, adding 100 mg of curcumin or 15 mg of TMX and stirring for 12 h at room temperature.

To understand the effect of the addition of hydrophilic polymers, uniaxial fibres were prepared using a PCL–Alg mixture loaded with Cur (PCL–Alg–Cur1). These nanofibers were prepared by mixing 8 mL of a 10% *w*/*v* PCL solution with 2 mL of a 0.1% *w*/*v* Alg solution in a mixture of glacial acetic acid, ethyl acetate and water in a 3:2:1 ratio, before adding the curcumin and stirring for 12 h at room temperature. The PCL–Alg (PCL–Alg–Cur2) and PCL–Gel (PCL–Gel–Cur) combinations, both loaded with curcumin, were used to make coaxial fibres. The nanofiber core was also produced with 10% *w*/*v* PCL solution loaded with curcumin and coated with a 0.1% *w*/*v* Alg or 8% *w*/*v* Gel solution. The solutions were taken to a Harvard Apparatus pHD 2000 injection pump connected to a Gamma High Voltage Research ES40 voltage generator. The pump used a metal needle with an internal diameter of 1 mm, with a distance of 20 cm between the tip of the injection needle and the collection plate.

### 2.3. Physicochemical Characterization

The starting materials and nanofibers were characterised using the following physicochemical techniques: absorption spectroscopy in the infrared region, X-ray diffraction and thermal analysis. In the infrared analysis, a Perkin Elmer spectrum GX spectrophotometer was used; the samples were prepared with KBr and readings were made in the 4000–400 cm^−1^ region. The crystallographic profile of both raw materials and fibres was obtained by X-ray diffraction. The X-ray diffraction pattern of the nanofibers was analysed using a Shimadzu XRD-700-X-Ray diffractometer, equipped with a copper tube and CuKα (λ = 1.5405 Å) radiation. Data were processed in the 2θ angles ranging from 4 to 60° and the scanning rate was 4θ min^−1^. Thermogravimetric (TG) profiling was performed using a TA Instruments SDT Q600 analyser, in N_2_ atmosphere with a constant flow rate of 50 mL.min^−1^, in the temperature range of 25–800 °C using a heating rate of 10 °C min^−1^. The morphology and diameter of the fibres were studied by scanning electron microscopy: portions of the fibres were deposited directly on copper sample holders inside the electrospinning equipment. The micrographs were taken with a FEG Quanta 2000 electron microscope using an acceleration voltage of 10 kV under reduced pressure.

### 2.4. In Vitro Release Profile

The electrospinning technique enables the uniform incorporation of bio-active molecules and nanoparticles in the nanofibrous polymeric matrix. The release profiles of uniaxial and coaxial nanofibers loaded with Cur and TMX were measured by incubating 1 cm^2^ fragments of each fibre in 2 mL of phosphate-buffered saline (PBS, pH7.4) with 0.01% sodium dodecyl sulphate (SDS) in a shaker thermostatted at 37 °C. The medium was replaced with the same amount of fresh medium after 1, 2, 4, 10, 24 and up to 300 h for quantification analysis. The amount of Cur released was quantified by UV-Vis spectroscopy at a wavelength of 427 nm, and the same was carried out for TMX at 365 nm.

The encapsulation efficiency of the nanofibers was determined indirectly, taking into account the percentage of release and taking into account the amount of curcumin (100 mg) or tamoxifen (15 mg) incorporated during the electrospinning process. The concentration of released curcumin and tamoxifen from nanofibers was calculated based on a curcumin and tamoxifen standard curve, respectively. The released drug (%) was estimated as the given equation:

Release (%) = (released drug)/(total drug) × 100

### 2.5. In Vitro Cytotoxicity Tests

For in vitro cytotoxicity analysis, an MCF-7 breast cancer cell line was obtained from frozen laboratory stock vials obtained from ATCC (Manassas, VA, USA). Those cells were cultured in a DMEM medium prepared with 1% (*v*/*v*) antibiotic and antifungal solution (penicillin 10,000 units/mL and streptomycin 10,000 μg/mL) supplemented with 10% foetal bovine serum (FBS) in 75 cm^2^ plastic bottles at 37 °C in a humidified atmosphere with 95% humidity and 5% CO_2_. Normal cells were isolated from 10 mL of peripheral blood from a healthy volunteer who had given informed consent. The blood was collected in tubes with heparin, and mononuclear cells (PBMCs) were obtained by Ficoll gradient. The tubes were centrifuged at 2.000 rpm for 5 min and then the buffy coat was removed with a sterile 2 mL pipette. This was gently added to a 15 mL tube with 2 mL of Ficoll Histopaque-1077, then centrifuged without brake for 20 min at 2.000 rpm. The white layer was recovered with a sterile 2 mL pipette and poured into a fresh tube containing 5 mL of 1 × PBS, then centrifuged at 2.500 rpm for 5 min. Then, 5 mL of PBS was added to the pellet, and it was centrifuged again. Immediately the new cell pellet was gently resuspended in a fresh 15 mL tube containing 5 mL of PB-MAX karyotyping medium with 100 µL of phytohemagglutinin (PHA M) and antibiotic and antifungal solution. The tube was stored at 37 °C and 5% CO_2_ for 24 h before treatment with the nanofibers. Then, PBMCs and MCF7 cells were seeded in duplicate in a 96-well culture plate at a density of 15,000/200 µL for 24 h. After, a 7 mm diameter circle of each of the nanofibers was added to a well and left for 1 to 6 days.

The cytotoxicity of all fabricated nanofibers was evaluated by the resazurin assay as an indirect measure of cell viability. Treatments can be monitored by taking several measurements of the same group of cells at different times, as resazurin is non-toxic [46,47]. The plates were removed from the incubator for a short time (5–10 min) to take the measurements and then returned. This test indicates the number of viable cells and the level of metabolic activity in a sample. The nanofiber specimens with TMX had a concentration of 16 µM and those with curcumin had a concentration of approximately 40 µM. Every day, a plate was removed from the incubator and the culture medium was removed; the wells were washed with 200 µL of PBS, and fresh serum-free medium with 4.4 µM resazurin was added, followed by additional incubation under the same initial conditions. After 4, 6 and 24 h, fluorescence intensity was measured at the emission wavelength of 595 nm and excitation wavelength of 535 nm using a cell imaging multimode microplate readers Cytation 3a [47,48]. Some MCF7 cells were also exposed under the same conditions described above, to concentrations of free TMX (0–50 nM) and others to curcumin (0–150 µM), and fluorescence intensity values were obtained at 4, 6, 24 and 30 h after the addition of resazurin.

For PBMCs, assays were incubated with the nanofibers for 24 h. The plates were then centrifuged at 2.000 rpm for 5 min and the medium was replaced with PB-MAX containing 4.4 µM resazurin, followed by a further incubation period under the same conditions. Fluorescence was measured in the same manner as described above after 4, 6, 24, 24, 30 and 48 h.

From the fluorescence intensity values obtained were subtracted the value produced by the reagent alone for all cases. The percent viability was calculated by comparing the data to the untreated group, which was assumed to have 100% viability. Cytotoxicity was calculated based on cell viability relative to this group: none ≥ 90%; mild = 60–90%; moderate = 30–59%; and severe ≤ 30% [49].

### 2.6. Statistical Analysis

The results were organised by cell type, treatment, and time of exposure to treatment and resazurin. The treatments were: no nanofiber, PCL, PCL–Cur, PCL–Alg–Cur1, PCL–Alg–Cur2, PCL–Gel–Cur and PCL–TMX. The percentage of viability was calculated and the Shapiro–Wilk normality test was applied to these data. All the data were normally distributed. The treatments were then compared using Student’s *t*-test for unpaired variables; MCF-7 without treatment was compared with all treatments: PCL vs. MCF7, PCL–Cur vs. MCF7, PCL–Gel–Cur vs. MCF7, PCL–Alg–Cur1 vs. MCF7, PCL–Alg–Cur2 vs. MCF7 and PCL–TMX vs. MCF7. Similarly, PBMCs were compared: PCL vs. PBMCs, PCL–Cur vs. PBMCs, PCL–Gel–Cur vs. PBMCs, PCL–Alg–Cur1 vs. PBMCs, PCL–Alg–Cur2 vs. PBMCs and PCL–TMX vs. PBMCs. In some figures, the level of significance is indicated as follows: a = *p* * < 0.05, b = *p* ** < 0.01, c = *p* *** < 0.001.

In addition, an ANOVA was applied to compare each cell type (MCF7 and PBMCs) without treatment to all when each treatment was applied. All the treatments showed statistically significant differences, but this test does not discriminate between groups. In addition, the Bonferroni test was used to compare each treatment with cells not exposed to nanofibers.

## 3. Results

### 3.1. Morphological Characterization

**Figure 1:**
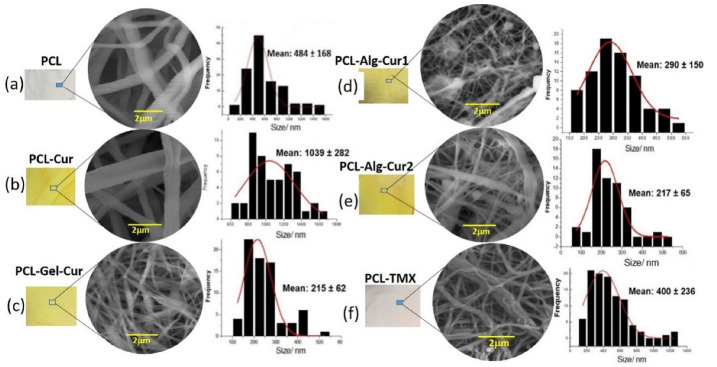
Morphology and diameter distribution of the nanofibers. SEM micrographs and diameter distribution histograms of uniaxial and coaxial PCL nanofibers: (**a**) PCL (**b**) PCL–Cur (**c**) PCL–Gel–Cur (**d**) PCL–Alg–Cur1 (**e**) PCL–Alg–Cur2 and (**f**) PCL–TMX.

The morphological characterization and geometrical evaluation of the fibres (size distribution by diameter and orientation) were studied by scanning electron microscopy (SEM). In general, the morphology and size of the fibres are influenced by different factors such as viscosity, polymer concentration and solvent type, and operational factors such as the distance between the needle and the collection plate, the current applied and the leakage flow of the polymer solution. Figure 1 shows the morphological characteristics and size distribution of different uniaxial PCL nanofibers alone and loaded with Cur and TMX (Figure 1a,b,f), uniaxial PCL–Alg nanofibers (Figure 1d), coaxial PCL–Alg (Figure 1e) and PCL–Gel (Figure 1c) nanofibers.

The micrographs and diameter distribution diagrams show the effect of both the polymer and drug on the morphology of the nanofibers. The uniaxial PCL–Cur nanofibers had a size distribution between 600 nm–1.2 µm and an average diameter of 1.03 µm with a uniform appearance, while the TMX-loaded fibres had an average diameter of 400 nm, also with a uniform morphology and no imperfections. This increase in the size of the curcumin-loaded nanofibers can be explained by several factors, one of them being the injection rate of the polymeric mixture used (3 mL/h), as different authors report that higher injection rates (above 0.5 mL/h) encourage the formation of larger drops at the point of the needle, which consequently lead to a larger diameter in the fibres formed. Another factor was the presence of a molecule with non-polar characteristics such as curcumin and the type of solvent used in the preparation of the polymeric mixture (methanol and dichloromethane), as this mixture has high relative permittivity values, which is a determining factor in the fibre formation process [50,51].

Uniaxial nanofibers with the presence of alginate (PCL–Alg–Cur1) showed a smaller size distribution pattern in the range of 150–550 nm, with an average size of 290 nm and a morphology with the formation of beads distributed throughout the nanofiber structure. This drastic decrease in particle size compared to the nanofibers that had only PCL may be due to the low injection rate of the polymeric mixture (1.5 mL/h) and the type of solvents used in the preparation of the solution, since the mixture of acetic acid and ethyl acetate allowed us to increase its polarity, and consequently, the charge density, improving the electrical conductivity of the droplet surface, thereby promoting the decrease in the diameter of the fibres formed [52]. Unlike the uniaxial fibre, the coaxial fibre with the presence of alginate (PCL–Alg–Cur2), besides having a size distribution between 100–500 nm with an average diameter of 215 nm, had a large number of defects in the overall network, which include alginate beads or spheres on the PCL-formed fibres. This may be due to the fact that the presence of alginate (in low concentrations) did not allow the proper formation of the coating fibres. This behaviour has already been reported by other authors who identify that there are problems in the formation of fibres in PCL blends with polymers such as alginate and chitosan, since these polymers have positive and negative charges that generate fibres with defects and agglomerates. Furthermore, in this specific case, the alginate solution was used as an external coating layer, and the low fibre formation capacity of this biopolymer can be explained by the rigidity of the molecule in aqueous solution [53,54,55].

For their part, the PCL–Gel–Cur coaxial fibres had a size distribution with diameters in the range of 100–500 nm and an average diameter of 215 nm (Figure 1c). The microphotograph shows the formation of some defects that can be explained by both the injection rate of the polymer (1.5 mL/h) and the volume of the polymer gelatine solution used in the mixture [56,57]. Another of the factors that affects the formation of nanofibers and could be a determinant in the preparation is the viscosity, since it is known that high viscosity values prevent the formation of smooth and uniform fibres. Additionally, the charge density is a parameter that is related both to conductivity and to the type of polymer and solvent chosen. Thus, natural polymers behave as polyelectrolytes, increasing the charge transport through the polymer solution, resulting in a higher voltage in the electric field, which prevents fibre formation. The type of solvent and polymer used directly affects this parameter.

### 3.2. Physicochemical Characterization of the Starting Materials and Nanofibers

It is known that the major disadvantage of synthetic polymers such as PCL is the lack of cell recognition signals. However, there are strategies to modify the surface characteristics of synthetic polymers to improve cell adhesion, proliferation, migration and differentiation, including blending these polymers with substances with hydrophilic characteristics and preferably with other polymers of natural origins [42]. Therefore, blending and/or combining PCL with polymers such as alginate, gelatine or chitosan has been shown to be a promising technique to promote cell recognition sites and the hydrophilicity of PCL [19,37,43].

**Figure 2:**
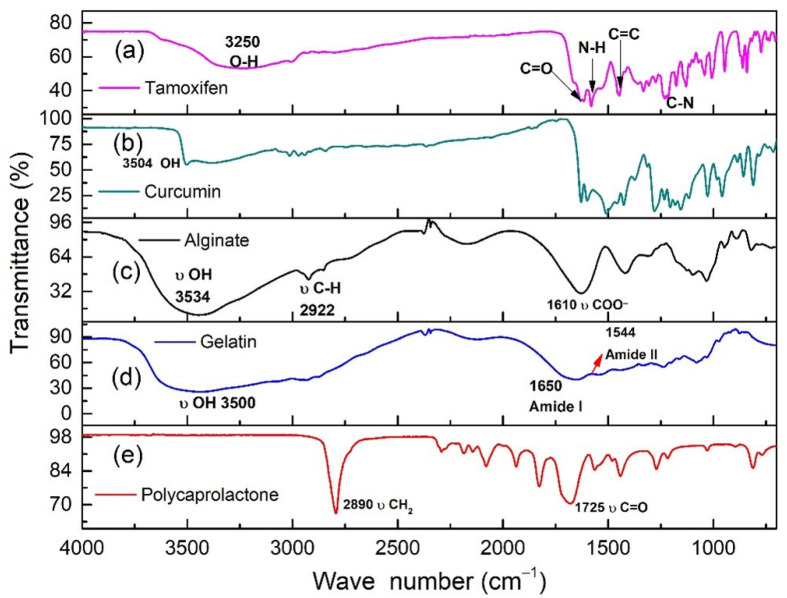
FTIR spectra of the materials used in the fabrication of the nanofibers. (**a**) TMX (**b**) curcumin (**c**) alginate (**d**) gelatine and (**e**) polycaprolactone.

To evaluate the type of interaction observed between PCL and the other components in the polymer nanofibers, the chemical and physicochemical characteristics of both the starting materials and the fabricated uniaxial and coaxial nanofibers were studied. In the case of the starting materials, the infrared spectra showed characteristic bands for each material. It can be seen in Figure 2a that TMX had a broad band at 3229 cm*^−^*^1^ due to the O-H functional group of the alcohol and phenolic groups, a characteristic C=O band at 1627 cm*^−^*^1^, an N-H band at 1575 cm*^−^*^1^, the C=C stretching band (reflecting ring vibrations) at 1453 cm*^−^*^1^, the C-N amino stretching bands at 1227 cm*^−^*^1^ and the C-O phenolic stretching band at 1174 cm*^−^*^1^. For Cur (Figure 2b), we observed a broad band at 3504 cm*^−^*^1^ attributed to OH functional groups, as well as bands at 1628 cm*^−^*^1^ corresponding to symmetrical vibrations of the C=C and C=O functional groups, a strong band at 1601 cm*^−^*^1^ related to symmetric vibrations of the C=C groups of the aromatic structures, a band at 1508 cm*^−^*^1^ corresponding to vibrations of the C=O and C=C groups and bands at 1028 and 958 cm*^−^*^1^ related to vibrations of the C-C-H and C-O groups, respectively [58,59,60].

In the case of alginate, there was a band at 3534 cm*^−^*^1^ corresponding to O-H groups, an intense band at 2922 cm*^−^*^1^ characteristic of asymmetric deformations of C-H_2_ groups and a strong band at 1610 cm*^−^*^1^ characteristic of COO- groups [61]. The gelatine spectrum presented a broad band at 3500 cm*^−^*^1^ characteristic of O-H groups, and bands at 1650 cm*^−^*^1^ and 1544 cm*^−^*^1^ corresponding to amide I and amide II functional groups, respectively [62]. The polycaprolactone spectrum showed strong bands at 2890 cm*^−^*^1^, characteristic of vibrations of C-H bonds in CH_2_ groups; and at 1725 cm*^−^*^1^, characteristic of deformations of C=O groups [63].

**Figure 3:**
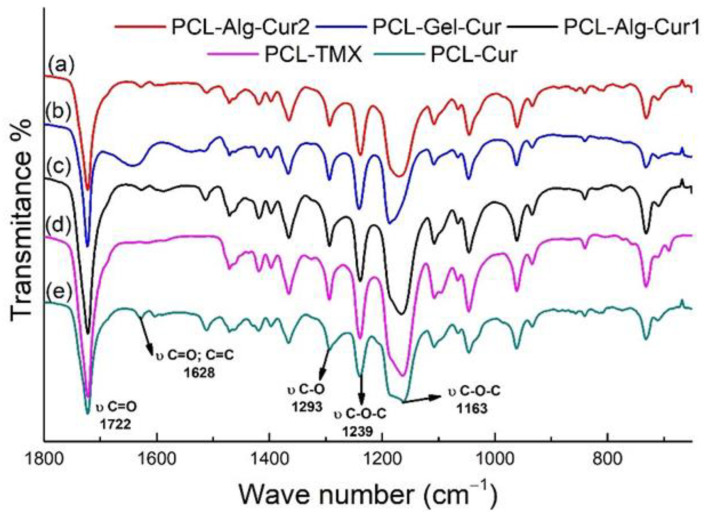
FTIR spectra of the nanofibers manufactured. (**a**) PCL–Alg–Cur2 (**b**) PCL–Gel–Cur (**c**) PCL–Alg–Cur1 (**d**) PCL–TMX and (**e**) PCL–Cur.

The IR spectra for the different nanofibers manufactured showed similar patterns (Figure 3) while de curcumina was different (Figure S1) as well as policaprolactona (Figure S2). Bands characteristic of the starting materials were observed in the prepared fibres: an intense band at 1722 cm*^−^*^1^, characteristic of PCL and typical of C=O group vibrations; a band at 1628 cm*^−^*^1^, corresponding to vibrations of C=O and C=C groups in curcumin; and bands at 1293, 1239 and 1163 cm*^−^*^1^, corresponding to vibrations of C-O and C-O-C groups characteristic of polycaprolactone [64]. This shows that both curcumin and tamoxifen are incorporated into the fibres and that the percentage of hydrophilic polymers (alginate and gelatine) is very low compared to that of PCL.

**Figure 4:**
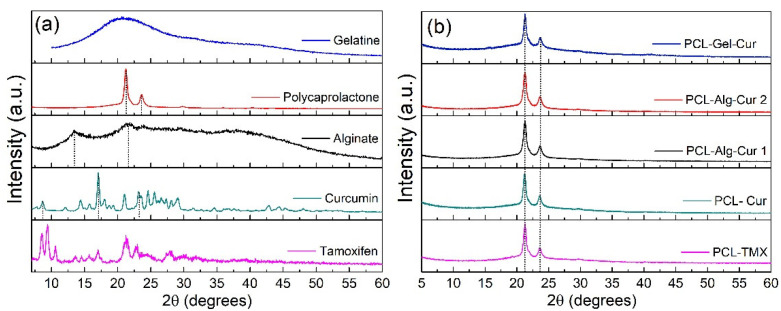
XRD diffractogram for (**a**) the starting materials and (**b**) the uniaxial and coaxial nanofibers. Arbitrary units (a.u.).

The diffractograms and XRD profile showed differences between the starting materials (Figure 4a) and the fabricated nanofibers (Figure 4b). It can be seen that curcumin had a characteristic polycrystalline pattern with peaks at 8.5, 17.5 and 23.1 degrees, respectively [65]. Alginate manifested an amorphous pattern specific to polymeric materials with broad peaks at 12.5° and 21.5° [65]. The diffractogram for PCL showed two peaks at 21.2 and 23.6, corresponding to the (110) and (200) crystallographic planes of this polymer [66]. Gelatine had an amorphous pattern with a broad peak characteristic of this biopolymer [67] and TMX presented low intensity peaks due to its polycrystalline structure, with the main peaks at 8.5, 9.3, 10.6, 17.0, 21.1 and 23.0 degrees, as previously reported in the literature [67,68,69].

For its part, Figure 4b presents the XRD diffractograms for the prepared uniaxial and coaxial fibres, which all showed a similar pattern with peaks at 21.2° and 23.6° corresponding to the PCL signals or profile. As can be seen, both the FITR and XRD data showed that the curcumin and TMX medications are not found on the nanofiber surface—they are encapsulated or inside the fibres—and that the hydrophilic polymers (Alg and Gel) that are found on the surface are in a very low proportion that do not chemically affect the composition of the nanofiber surface.

**Figure 5:**
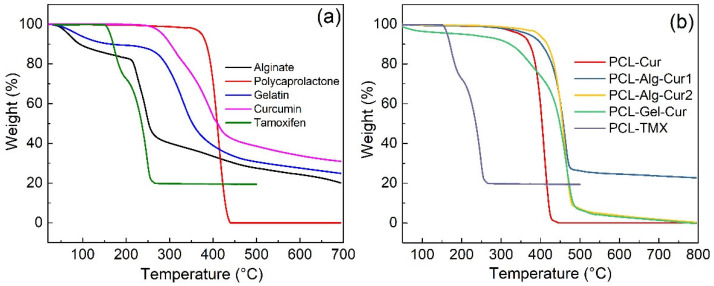
Thermogravimetric curves of (**a**) the materials used in the manufacture of the nanofibers, and (**b**) the uniaxial and coaxial nanofibers.

There were some differences between the thermogravimetric curves of the starting materials (Figure 5a) and the fabricated uniaxial and coaxial PCL nanofibers (Figure 5b). For the starting materials, the mass loss events and thermal decomposition profiles for curcumin, tamoxifen, polycaprolactone, alginate and gelatine are similar to those described in the literature. Polycaprolactone showed a stable profile in the 25 °C–372 °C range, after which it initiates thermo-decomposition processes, as reported in the literature [70]. Curcumin showed a stable profile between 25 °C and 264 °C range, after which the increasing temperature leads to thermal degradation events that are related to the degradation of the molecule’s own benzene rings [71]. Sodium alginate and gelatine showed less stable profiles characterised by a first mass loss between room temperature and 208 °C and 230 °C range, respectively, and a second event between 250 °C and 360 °C—events related to the breakage of C-H and C-O-H glycosidic bonds [72,73]. For the case of nanofibers (Figure 4b), it can be observed that PCL–Cur, PCL–Alg–Cur1 and PCL–Alg–Cur2 had a stable profile between 25 °C and 317 °C, with PCL–Alg–Cur1 and PCL–Alg–Cur2 fibres showing the highest thermal stability, possibly due to the presence of a polymer such as alginate, which can present higher intramolecular cross-linking between its monomers. In the case of PCL–Gel–Cur nanofibers, although there is a mass loss of 4% in the 25–85 °C range, followed by a stable pattern in the 85–255 °C range, further along the thermogravimetric profile, there is evidence of higher thermal stability compared to the PCL–Cur and PCL–TMX fibres, most probably due to the presence of gelatine, which protects the active molecule from degradation.

### 3.3. In Vitro Release Profile

**Figure 6:**
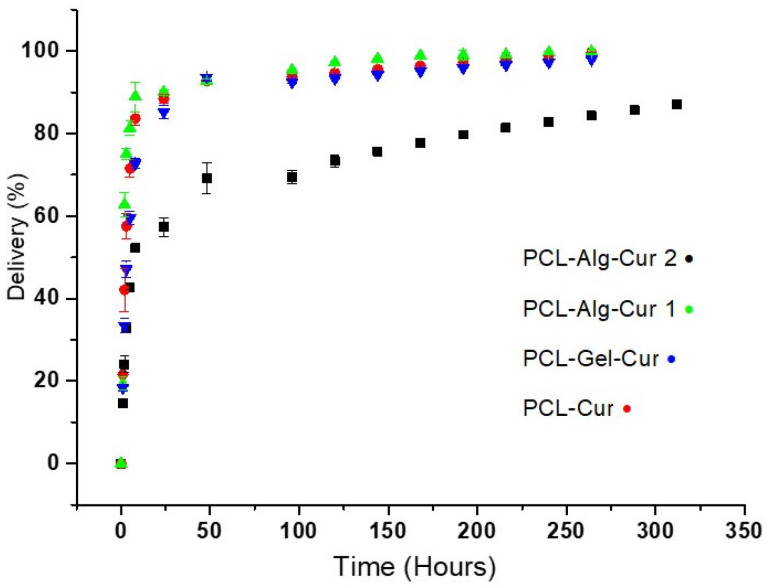
Release profile of curcumin (Cur) in vitro from uniaxial and coaxial nanofibers.

Figure 6 shows the release profile of the curcumin loaded on the uniaxial and coaxial nanofibers, in which two release domains are observed. The uniaxial fibres demonstrate a burst effect in the first 8 h, during which approximately 80% of the curcumin is released. During the second period, the profile stabilises and extends the release of the encapsulated curcumin in a controlled manner until 95% of the total is released after a period of up to 264 h (11 days). On the other hand, the PCL–Alg–Cur2 fibre released approximately 50% of curcumin in the first 8 h and maintained its release for more than 300 h (14 days), reaching only 80% of the encapsulated curcumin.

**Figure 7:**
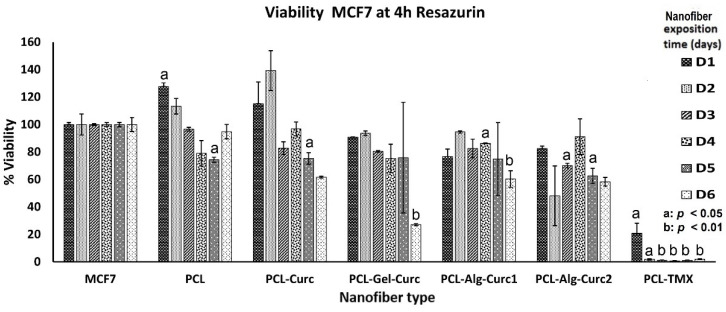
Viability percentage of MCF-7 cells treated with the different nanofibers for six days. The reduction in resazurin to resorufin was measured at 4 h. The student’s *t*-test was used to establish significance.

Prolonged release kinetics are common in hydrophobic molecules contained on hydrophobic surfaces such as polycaprolactone. However, the presence of hydrophilic surfaces such as alginate or gelatine promote faster release kinetics with a pronounced burst effect, such as that showed in PCL–Alg–Cur1 and PCL–Gel nanofibers (Figure 7). This behaviour was described by other authors [74,75]. In general, curcumin has a better solubility behaviour in alkaline pH due to the deprotonation of the hydroxyl groups [76]. Additionally, when using the phosphate buffer at pH 7.40, the crosslinking network of alginate and gelatine can be broken and can facilitate the penetration of water, which facilitates the diffusion of curcumin in the medium [77]. Another possible explanation for the higher release rate in the phosphate buffer could be related to the lower interaction of the carboxylic groups of hydrophilic polymers with the phosphate buffer, which allows the network to be loose (erosion-diffusion effect), which may facilitate the leaching of curcumin from the network to the medium of dissolution [78]. Another factor that allowed the complete release of curcumin was the modification of the release medium with SDS surfactant. The non-modification of the release medium generates slow release kinetics with low curcumin release percentages in similar matrices [79,80]. This release medium modification strategy is a tool that has been used for other types of hydrophobic molecules similar to curcumin, using polycaprolactone as a matrix [80]. The 50% TMX release was observed in the first 2 h; from this moment, it was progressive and reached 100% at 14 h (data not showed).

These results implied that calcium alginate and gelatine not only protected curcumin but also controlled curcumin release under in vitro conditions.

### 3.4. In Vitro Cytotoxicity

**Figure 8:**
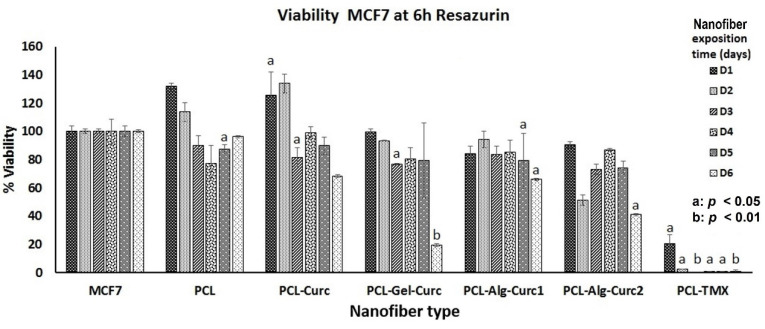
Viability percentage of MCF-7 cells treated with the different nanofibers for six days. The reduction in resazurin to resorufin was measured at 6 h. The student’s *t*-test was used to establish significance.

**Figure 9:**
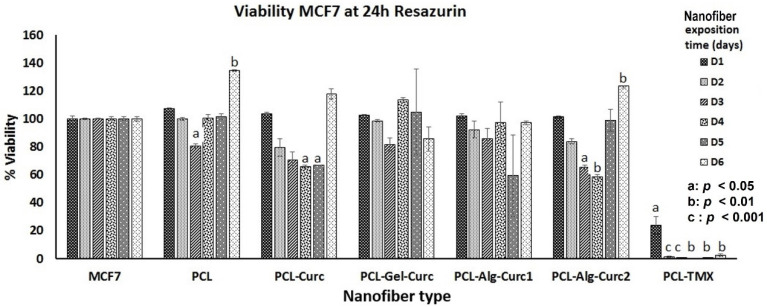
Viability percentage of MCF-7 cells treated with the different nanofibers for six days. The reduction in resazurin to resorufin was measured at 24 h. The student’s *t*-test was used to establish significance.

The assay used to determine the cytotoxic effect of uniaxial and coaxial nanofibers is based on the fact that resazurin, a blue dye, is metabolised by mitochondrial enzymes in the cells, which transform it into the fluorescent pink resorufin, which the cells release into the culture medium. PCL–TMX showed higher cytotoxicity against MCF-7 cells than the other nanofibers (Figure 7, Figure 8 and Figure 9); 20% cell viability was observed on the first day, compared to 100% and 127% viability observed in untreated and PCL incubated cells, respectively. After the second day of incubation, the PCL–TMX nanofibers reduced the viability drastically to values between 1.8 and 0.6%, with statistical significance in all cases.

On the contrary, those incubated with PCL had an increase in viability on the first two days of 127% and 115%, respectively, then remained very close to the untreated cells, and on day 6, they reached values of 134%, showing that the PCL platform slightly stimulates viability. The differences between untreated cells and those with PCL were statistically significant for day 1 (4 h Rsz) incubation, day 4 (24 h Rsz), day 5 (6 h Rsz) and day 6 (24 h Rsz), with values of *p* = 0.016, *p* = 0.043, *p* = 0.017 and *p* = 0.032, respectively. Curcumin fibres reduced the viability of MCF-7 after the second day of incubation. Curcumin-containing fibres reduced the metabolic activity of MCF-7 after the second day of incubation, but the cells showed a moderate recovery at 24 h after removal of the nanofiber. Coaxial nanofibers with curcumin using Alg (PCL-Arg-Cur2) decreased viability similarly to PCL–Cur nanofibers, although after 6 days of exposure, the cells recovered their metabolic activity and showed viability above 100% (Figure 9). All data of the cytotoxicity tests with the respective statistical analysis are showed in Table S1.

The PCL–Cur nanofibers reduced the viability more than the other nanofibers with curcumin until day four of incubation, but an increase was observed particularly on day six, when the values were similar to those of the untreated cells (Figure 9).

Free curcumin at the highest concentration (150 µM) drastically reduced MCF-7 viability during the first six hours of exposure, although all concentrations used were cytotoxic after two days of treatment. Meanwhile, free TMX severely reduced viability at the highest concentration from the third day of treatment. This showed that the action of free TMX on viability decrease was faster when it was released by the nanofiber. Contrary to what happens with free curcumin, whose action is more severe from the second day of exposure while encapsulated in the nanofibers, a moderate decrease was observed from the third day.

**Figure 10:**
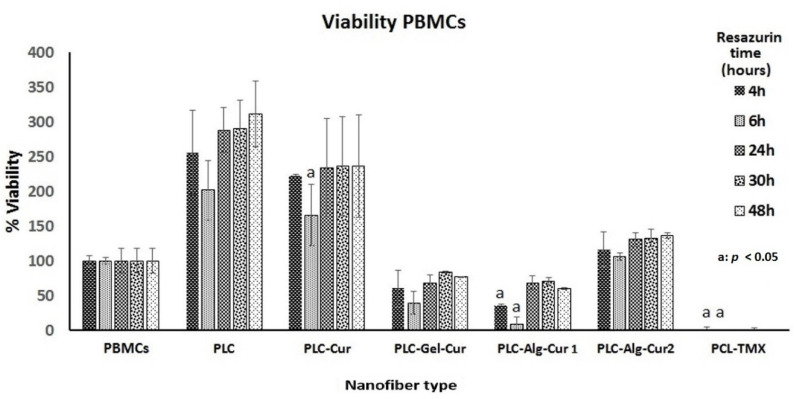
Viability percentage of PBMCs cells treated with the different nanofibers for 24 h. The reduction in resazurin to resorufin was measured at 24 h. The student’s *t*-test was used to establish significance.

PBMCs cannot be kept in culture for a long time as they are normal cells, so they were incubated for one day with the nanofibers, and fluorescence was measured between 4 and 48 h after adding resazurin. It was observed that PCL and PCL–Cur strongly stimulated the viability of these cells, while PCL–Gel–Cur decreased viability at 4 and 6 h, but after 24 h–36 h, the cells were able to recover and the effect was slight. PCL–Alg–Cur2 did not affect cell viability (Figure 10).

**Figure 11:**
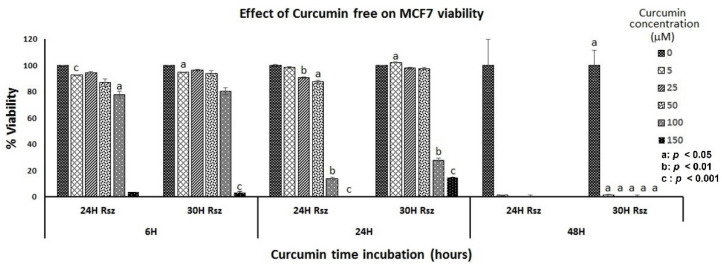
Viability percentage of MCF7 cells in the presence of free curcumin: 15,000 MCF7 cells were treated for 6, 24 and 48 h with different concentrations of free curcumin (0, 5, 25, 50, 100 and 150 µM). The reduction in resazurin to resorufin was measured at 4, 6, 24 and 30 h.

**Figure 12:**
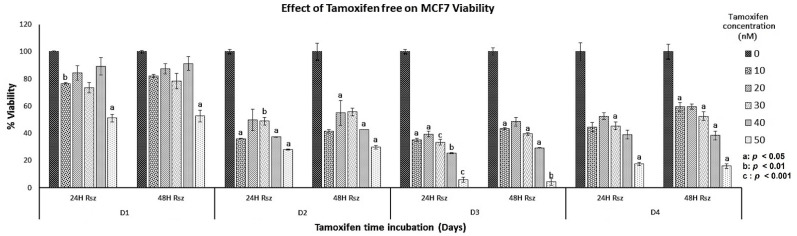
Viability percentage of MCF7 cells in free tamoxifen: 15,000 MCF7 cells were treated for 3 days with different concentrations of tamoxifen-free (0–50 nM). The reduction in resazurin to resorufin was measured after 36 h of incubation.

MCF-7 cells were cultivated with free concentrations of curcumin of 0–150 µM, and tamoxifen of 0–100 nM; a similar effect was observed in the viability (Figure 11 and Figure 12).

## 4. Conclusions

It is well known that nanometric-scale biomaterials, especially natural or synthetic polymer nanofibers, are used in current research for the prevention, treatment, and diagnosis of diseases such as cancer, and local delivery systems for drugs or active substances.

In addition to presenting characteristics of biocompatibility and biodegradability, these systems must allow the controlled release of active agents and be similar to the native extracellular matrix of human tissues and cells [41,81,82].

Thus, local delivery systems have the advantage over systemic therapy of being able to provide continuous delivery of drugs at higher concentrations and directly to the target site. Benefits of these systems include improved patient compliance, reduced toxic effects and systemic complications [83]. Therefore, the development of locally delivered nanofibers with different physicochemical characteristics may be a promising opportunity for the efficient treatment of solid tumours.

To the best of our knowledge, no comparative studies have reported on uniaxial and coaxial nanofibers containing the combination of PCL with alginate and/or gelatine, and the effect on cell viability of the MCF7 cell line and low toxicity in PBMCs cells.

Nanofibers with TMX and curcumin were effectively produced by electrospinning and showed a high release rate in the first 24 h after coming into contact with a suitable medium.

Both chemicals reduced cell viability—TMX reduced cell viability more rapidly and severely when it was released from the nanofibers than when it was free, while free curcumin at high concentrations acted faster and more severely than when released by nanofibers.

PCL–Alg–Cur2 did not affect the cell viability of PBCMs cells, so this may be the best vehicle to transport curcumin. TMX-free was shown to severely affect the viability of PBMCs.

Different studies have evidenced that core–shell curcumin nanofibers have antibacterial properties. However, few studies have showed the advantages of adding hydrophilic polymers to PCL structure and the modulate release effect on toxicity in MCF7 cancer cells. Likewise, no studies related to nanofibers determine the effect of these biomaterials on PBMC cells, since most of them refer to fibroblast cell lines (L929, 3T3, human fibroblasts, etc.).

On the other hand, the studies related to the antiproliferative activity of curcumin have not compared its effects with tamoxifen (as a reference molecule); the present work showed that despite the high effectiveness of tamoxifen nanofibers on the MCF7 cell line, there is high PBMC cell toxicity. In this sense, the PCL–Alg–Cur and PCL–Gel–Cur nanofibers become an effective, non-toxic, antiproliferative local alternative for breast cancer treatment.

## Figures and Tables

**Table 1:** General conditions for preparing the nanofibers.

NANOFIBER	Abbreviation	Type	Solution PCL (Final %)	Solution Alginate (Final %)	Solution Gelatine (Final %)	CUR	TMX
						(mg)	(mg)
PCL	PCL	Uniaxial	100	-	-	-	-
PCL–Curcumin	PCL–Cur	Uniaxial	100	-	-	100	-
PCL–Tamoxifen	PCL–TMX	Uniaxial	100	-	-	-	15
PCL–Alginate–Curcumin	PCL–Alg–Cur1	Uniaxial	80	20	-	100	-
PCL–Alginate-Curcumin	PCL–Alg–Cur2	Coaxial	80	20	-	100	-
PCL–Gelatine–Curcumin	PCL–Gel–Cur	Coaxial	50	-	50	100	-

## Data Availability

The data that support the findings of this study are available from the corresponding authors upon reasonable request.

## Supplementary Materials

The following supporting information can be downloaded at: https://www.mdpi.com/article/10.3390/nano12193348/s1, Figure S1: IR spectrum of curcumin 1700–600 cm^−1^; Figure S2: IR spectrum of policaprolactona 1800–650 cm^−1^. Table S1: Data of the cytotoxicity tests with the respective statistical analysis.
