# Supplementary material for: Uniaxial and Coaxial Nanofibers PCL/Alginate or PCL/Gelatine Transport and Release Tamoxifen and Curcumin Affecting the Viability of MCF7 Cell Line
Source: Nanomaterials (Basel). 2022 Sep 26;12(19):3348. doi: 10.3390/nano12193348 (PMC9565524; doi:10.3390/nano12193348)
Supplement: Supplementary file 1 [file nanomaterials-12-03348-s001.zip › Figure S1 and Figure S2.pdf]

nanomaterials

Supplementary Materials

Uniaxial and Coaxial Nanofibers PCL/Alginate or PCL/Gelatine Transport and Release Tamoxifen and Curcumin Affecting the Viability of MCF7 Cell Line

Diego Fernando Suárez [1], Ana Delia Pinzón-García [1], Rubén Darío Sinisterra [1], Anderson Dussan [2], Fredy Mesa [2] and Sandra Ramírez-Clavijo [3,*]

[1] Chemistry Department, Instituto de Ciências Exatas, Universidade Federal de Minas Gerais, Av. Presidente Antônio Carlos 6627, Belo Horizonte 31270-901, MG, Brazil
[2] Departamento de Física, Grupo de Materiales Nanoestructurados y sus Aplicaciones, Universidad Nacional de Colombia, Bogotá 110011, Colombia
[3] Department of Biology, Grupo Ciencias Básicas Médicas, Faculty of Natural Science, Universidad del Rosario, Bogotá 110311, Colombia
* Correspondence: sandra.ramirez@urosario.edu.co

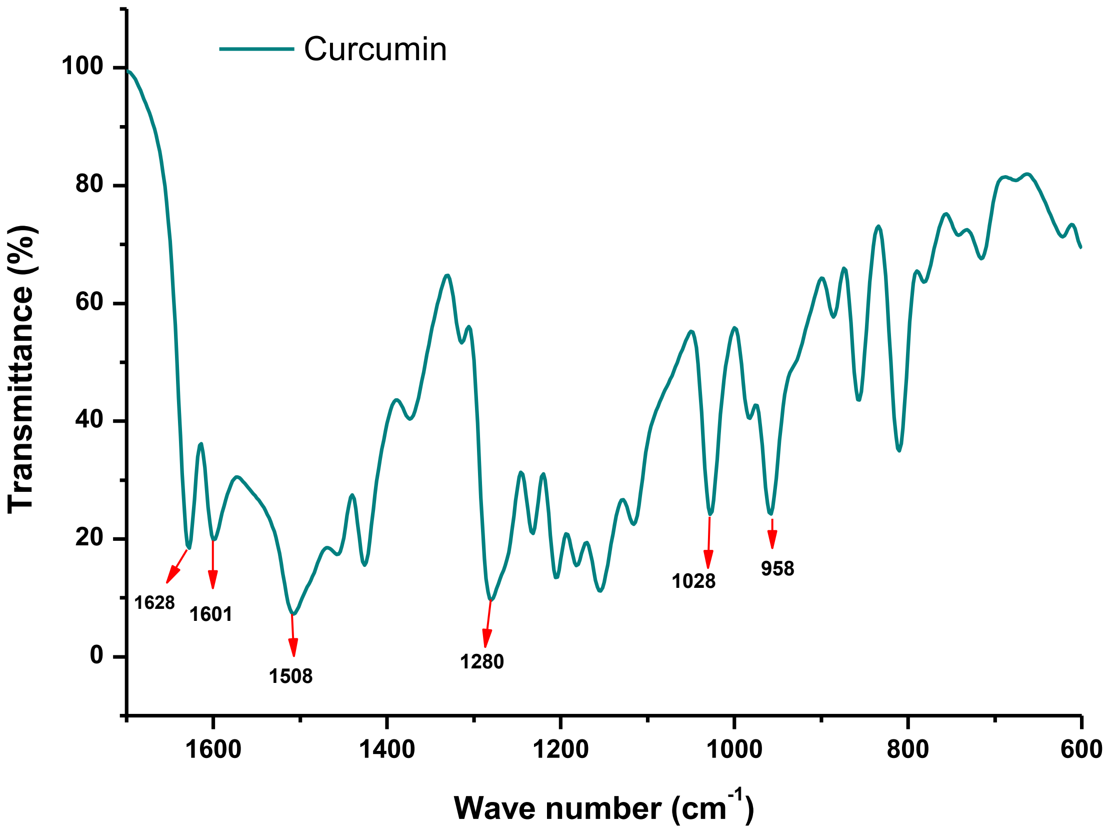

1628 cm^{-1}	1601 cm^{-1}	1508 cm^{-1}	1280 cm^{-1}	1028 cm^{-1}	958 cm^{-1}
υ C=O ,C=C	υ C=C	υ C=C; C=O	υ C=CH	υ C-C-H; CH_3	υ C-O

Figure S1. IR spectrum of curcumin 1700–600 cm^{-1}.

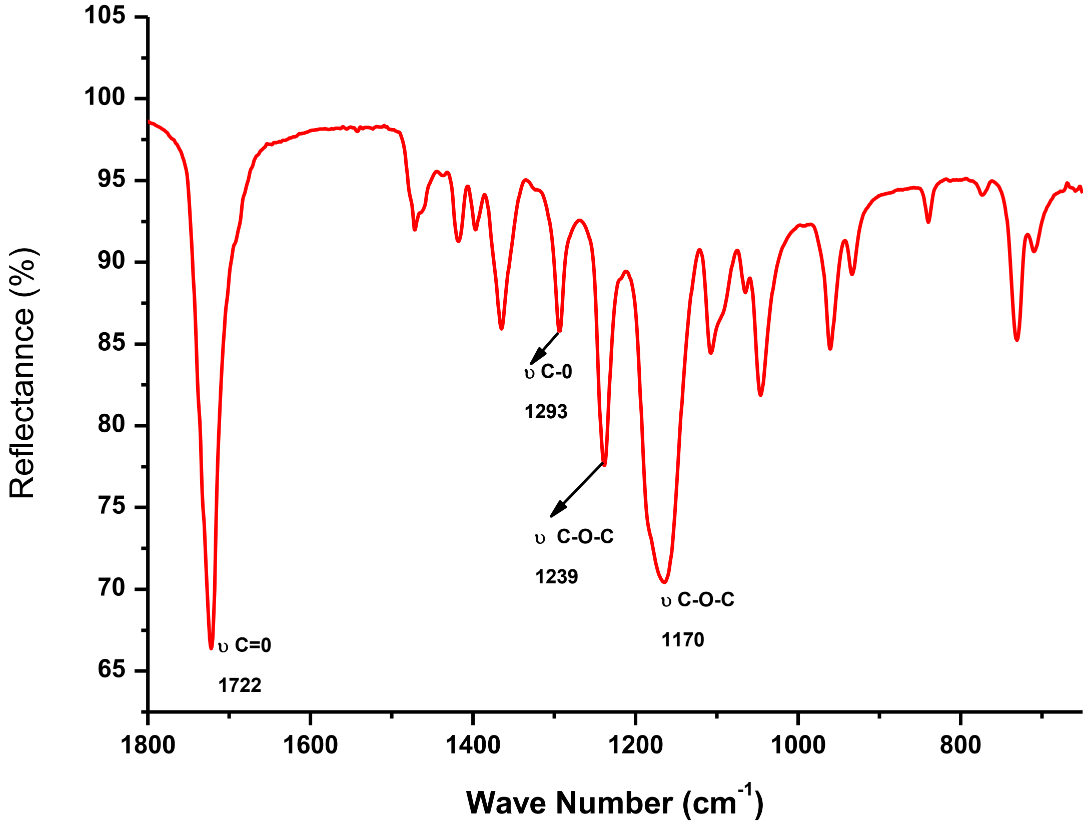

1722 cm^{-1}	1293 cm^{-1}	1239 cm^{-1}	1170 cm^{-1}
υ C=O	υ C-O	υ C-O-C	υ C-O-C

Figure S2. IR spectrum of policaprolactona 1800–650 cm^{-1}.
